# Deletion of hematopoietic Dectin-2 or CARD9 does not protect against atherosclerotic plaque formation in hyperlipidemic mice

**DOI:** 10.1038/s41598-019-40663-x

**Published:** 2019-03-13

**Authors:** Kathrin Thiem, Geerte Hoeke, Susan van den Berg, Anneke Hijmans, Cor W. M. Jacobs, Enchen Zhou, Isabel M. Mol, Maria Mouktaroudi, Johan Bussink, Thirumala D. Kanneganti, Esther Lutgens, Rinke Stienstra, Cees J. Tack, Mihai G. Netea, Patrick C. N. Rensen, Jimmy F. P. Berbée, Janna A. van Diepen

**Affiliations:** 1 Department of Internal Medicine and Radboud Institute for Molecular Life Sciences, Radboud university medical center, Nijmegen, The Netherlands; 2 Department of Medicine, Div. of Endocrinology, Leiden University Medical Center, Leiden, The Netherlands; 3 Einthoven Laboratory for Experimental Vascular Medicine, Leiden University Medical Center, Leiden, The Netherlands; 4 Department of Internal Medicine, National and Kapodistrian University of Athens, Medical School, Athens, Greece; 5 Dept. of Radiation Oncology, Radboud University Medical Center, Nijmegen, The Netherlands; 6 Department of Immunology, St. Jude Children’s Research Hospital, Memphis, TN USA; 7 Department of Medical Biochemistry, Div. of Experimental Vascular Biology, Academic Medical Center, University of Amsterdam, Amsterdam, The Netherlands; 8 Institute for Cardiovascular Prevention, Ludwig Maximilians University of Munich, Munich, Germany; 9 Div. of Human Nutrition, Wageningen University, Wageningen, The Netherlands; 10 Department for Genomics & Immunoregulation, Life and Medical Sciences Institute (LIMES), University of Bonn, Bonn, Germany

## Abstract

Inflammatory reactions activated by pattern recognition receptors (PRRs) on the membrane of innate immune cells play an important role in atherosclerosis. Whether the PRRs of the C-type lectin receptor (CLR) family including Dectin-2 may be involved in the pathogenesis of atherosclerosis remains largely unknown. Recently, the CLR-adaptor molecule caspase recruitment domain family member 9 (CARD9) has been suggested to play a role in cardiovascular pathologies as it provides the link between CLR activation and transcription of inflammatory cytokines as well as immune cell recruitment. We therefore evaluated whether hematopoietic deletion of Dectin-2 or CARD9 reduces inflammation and atherosclerosis development. Low-density lipoprotein receptor (*Ldlr*)-knockout mice were transplanted with bone marrow from wild-type, *Dectin-2-* or *Card9-*knockout mice and fed a Western-type diet containing 0.1% (w/w) cholesterol. After 10 weeks, lipid and inflammatory parameters were measured and atherosclerosis development was determined. Deletion of hematopoietic Dectin-2 or CARD9 did not influence plasma triglyceride and cholesterol levels. Deletion of hematopoietic Dectin-2 did not affect atherosclerotic lesion area, immune cell composition, *ex vivo* cytokine secretion by peritoneal cells or bone marrow derived macrophages. Unexpectedly, deletion of hematopoietic CARD9 increased atherosclerotic lesion formation and lesion severity. Deletion of hematopoietic CARD9 did also not influence circulating immune cell composition and peripheral cytokine secretion. Besides a tendency to a reduced macrophage content within these lesions, plasma MCP-1 levels decreased upon WTD feeding. Deletion of hematopoietic Dectin-2 did not influence atherosclerosis development in hyperlipidemic mice. The absence of CARD9 unexpectedly increased atherosclerotic lesion size and severity, suggesting that the presence of CARD9 may protect against initiation of atherosclerosis development.

## Introduction

Inflammation has been recognized as a key contributor to the development of atherosclerosis^1^. During the onset and progression of arterial lesion formation, monocytes infiltrate into the developing plaque area where they differentiate into different macrophage subsets. Pro-inflammatory macrophages cause a chronic state of inflammation through lipid accumulation and plaque destabilization whereas anti-inflammatory macrophages contribute to tissue repair, remodeling and plaque stabilization^2,3^.

Pattern recognition receptors (PRRs) play a key role in the innate immune response by recognizing a variety of exogenous infectious ligands and endogenous damage-associated molecules. Upon activation, PRRs induce the production of cytokines and other immune mediators that modulate inflammation and immunity. Toll-like receptors (TLRs), nucleotide-binding oligomerization domain (NOD)-like receptors (NLRs), Rig-I helicases and C-type Lectin receptors (CLRs) are the main families of PRRs that are expressed on cells of the innate immune system. Ample evidence shows that TLRs play a determinant role in the initiation and development of atherosclerosis^2,4^. Also, NLRs such as the NLR family pyrin domain containing 3 (NLRP3) inflammasome are involved in the development of atherosclerosis^5^.

However, whether CLRs are involved in atherosclerosis development is largely unknown. CLRs contain a carbohydrate recognition domain to recognize carbohydrates, but also non-carbohydrate ligands such as proteins and lipids^6,7^. The CLR family comprises various members, including Dectin-1 (CLEC7A), Dectin-2 (CLEC6A), and Mincle (CLEC4E). CLRs signal via recruitment to spleen tyrosine kinase (SYK) and subsequently via a complex that consists of the caspase recruitment domain family member 9 (CARD9), B-cell CLL/lymphoma 10 (BCL10) and mucosa-associated lymphoid tissue lymphoma translocation protein (MALT1). Signaling through this complex activates transcription factors such as nuclear factor-κB (NF-κB), thereby inducing transcription of *e.g*. interleukin (IL-)6 and tumor necrosis factor (TNF)α^6–8^, pro-inflammatory cytokines that have been implicated in atherosclerotic plaque progression^9^.

The role of the individual CLRs Dectin-1 and Mincle on atherosclerosis development have been studied using bone marrow (BM) transplantation experiments in low density lipoprotein receptor knockout (*Ldlr*^−/−^) mice. While hematopoietic Dectin-1-deficiency did not protect from atherosclerosis development^10^, deletion of hematopoietic Mincle reduced atherosclerotic lesion size and lipid accumulation within the plaque^11^. The role of Dectin-2 or downstream CLR signaling via CARD9 in atherosclerosis development has not been studied. However, Dectin-2 is highly expressed on macrophages, a cell type that is infiltrating the plaque and driving its formation^12^. CARD9 is involved in monocyte accumulation and pro-inflammatory cytokine secretion^13^. In addition, CARD9 mediates inflammation in macrophages *in vitro*, induced by oxLDL immune complexes^14^ or signals released by necrotic smooth muscle cell^14,15^. A pro-inflammatory response induced by CLR signaling via CARD9 could therefore potentially aggravate atherosclerosis development^13^. Hence, we hypothesize that activation of Dectin-2 and CARD9 aggravate inflammation and thereby enhance atherosclerosis development.

Therefore, this study aimed to decipher whether deletion of hematopoietic Dectin-2 or CARD9 reduces inflammation and atherosclerosis development. To this end, *Ldlr*^−/−^ mice were reconstituted with BM cells from control wild-type (WT) mice, *Dectin-2*- or *Card9-*knockout mice and, after recovery, fed a Western-type diet (WTD) containing 0.1% cholesterol to induce atherosclerosis. Our data show that deletion of hematopoietic Dectin-2 or CARD9 does not influence lipid or inflammatory parameters. In contrast to our hypothesis, deletion of hematopoietic Dectin-2 did not influence atherosclerosis development and plaque composition. Interestingly, deletion of hematopoietic CARD9 increased atherosclerotic lesion size and tended to reduce macrophage content within the atherosclerotic lesions.

## Results

### Deletion of hematopoietic Dectin-2 or CARD9 did not influence plasma lipids

**Figure 1:**
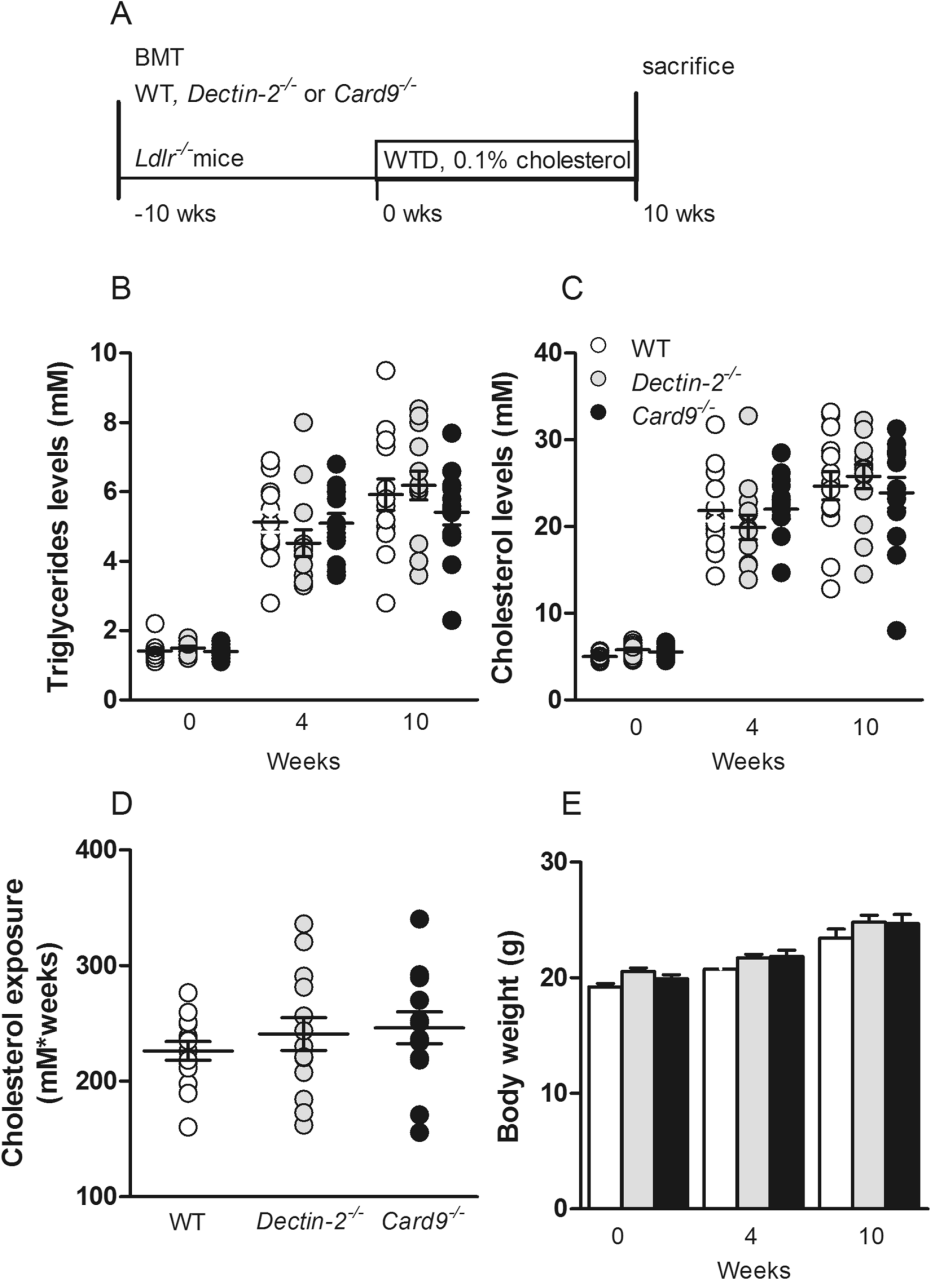
Deletion of hematopoietic Dectin-2 or CARD9 does not influence meta bolic parameters. (**A**) Lethally irradiated *Ldlr*^−/−^ mice were reconstituted with control (WT), *Dectin-2*^−/−^ or *Card9*^−/−^ BM. After 10 weeks of recovery, mice were fed a Western-type diet containing 0.1% cholesterol. Just before and after 4 and 10 weeks of WTD feeding, (**B**) triglyceride and (**C**) cholesterol levels were measured in plasma. (**D**) Total cholesterol exposure were determined at the end of the study and (**E**) body weight before and after 4 and 10 weeks of WTD feeding. Data are presented as mean ± SEM. n = 13–14/group.

To investigate the contribution of CLR signaling to atherosclerotic development, *Ldlr*^−/−^ mice were transplanted with BM from WT, *Dectin-2*^−/−^ or *Card9*^−/−^ mice and, after 10 weeks of recovery, received a WTD for 10 weeks (Fig. 1A). Deletion of hematopoietic Dectin-2 or CARD9 did not influence plasma triglyceride levels (Fig. 1B), cholesterol levels (Fig. 1C), and cholesterol exposure during the 10 weeks of WTD feeding (Fig. 1D) as compared to the WT-transplanted control mice. Also, body weight throughout the study was similar between the genotypes (Fig. 1E).

### Deletion of hematopoietic CARD9, but not Dectin-2, increased atherosclerotic plaque formation

**Figure 2:**
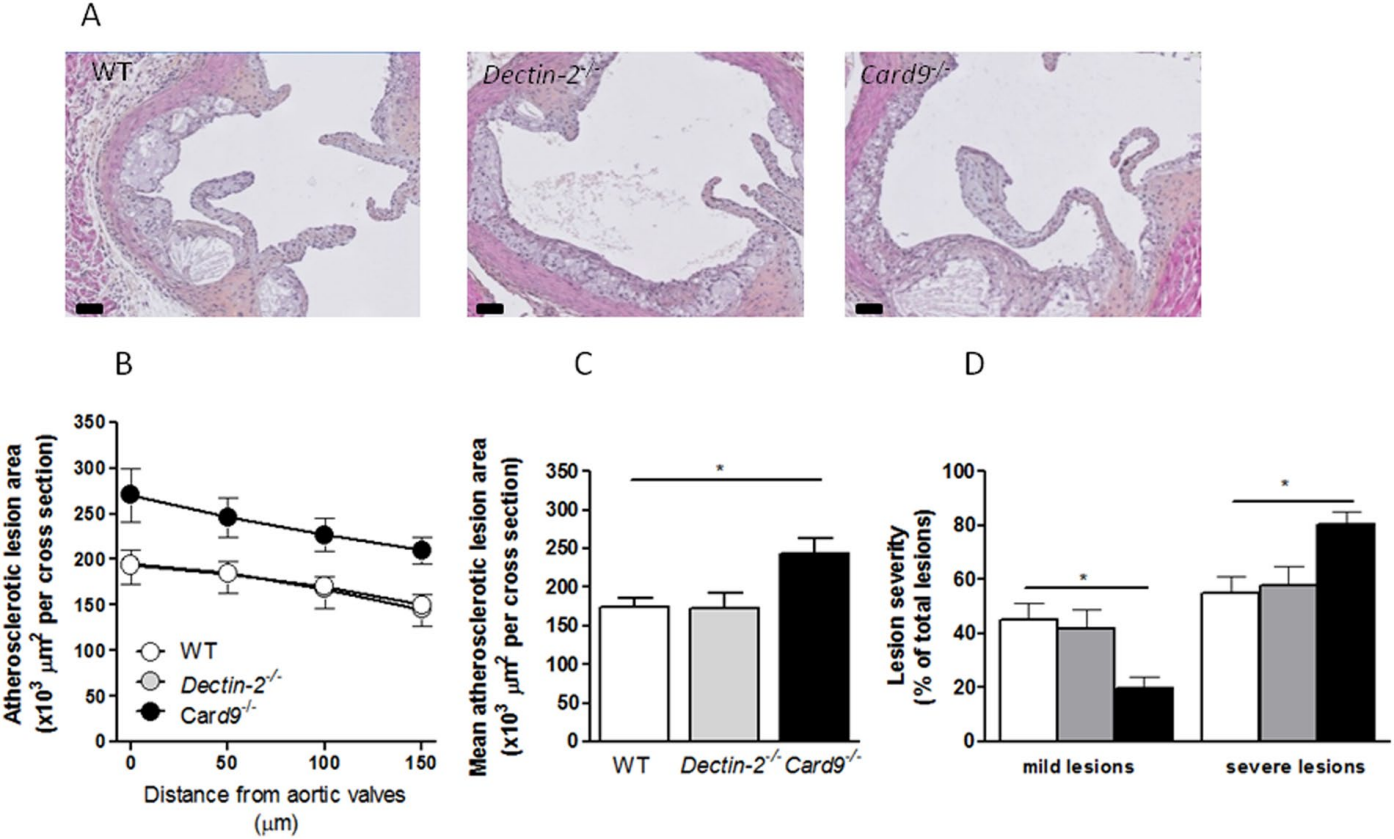
Deletion of hematopoietic CARD9, but not Dectin-2, increased atherosclerotic plaque formation. After 10 weeks of Western-type diet (WTD) feeding, hearts were collected from *Ldlr*^−/−^ mice transplanted with BM from control (WT), *Dectin-2*^−/−^ or *Card9*^−/−^ and aortic roots were analyzed by Immunohistochemistry. (**A**) Aortic valves were stained with hematoxylin-phloxine-saffron (HPS) and representative pictures are shown (scale bar represents 100 μm). (**B**) Total atherosclerotic lesion area was assessed in 4 sections of the aortic root and (**C**) average lesion area was calculated. (**D**) Lesion severity of was accessed following the guidelines of the American Heart Association adapted to mice and shown as percentage of total lesions. Data for atherosclerotic plaque composition are presented as mean ± SEM. n = 13–14/group. *p < 0.05.

First, atherosclerosis development in the different genotypes was assessed in the aortic root of the heart (Fig. 2). Deletion of hematopoietic Dectin-2 did not influence atherosclerotic lesion size as compared to control mice (Fig. 2A–C). Unexpectedly, deletion of hematopoietic CARD9 increased the atherosclerotic lesion area as compared to control mice (Fig. 2A–C). The increased lesion area coincided with more advanced lesion progression in hematopoietic deletion of CARD9 reflected by a shift towards fewer mild lesions (Fig. 2D; 20% vs. 45% p < 0.05) and more severe lesions (Fig. 2D; 80% vs. 55%, p < 0.05) compared to control mice. Hematopoietic deletion of Dectin-2 did not alter the percentages of mild (42%) and severe (58%) lesions as compared to control mice.

### Hematopoietic deletion of CARD9, decreased plasma MCP-1 levels, tended to decrease macrophage content but does not altered the distribution of M1/M2 markers in the plaque

**Figure 3:**
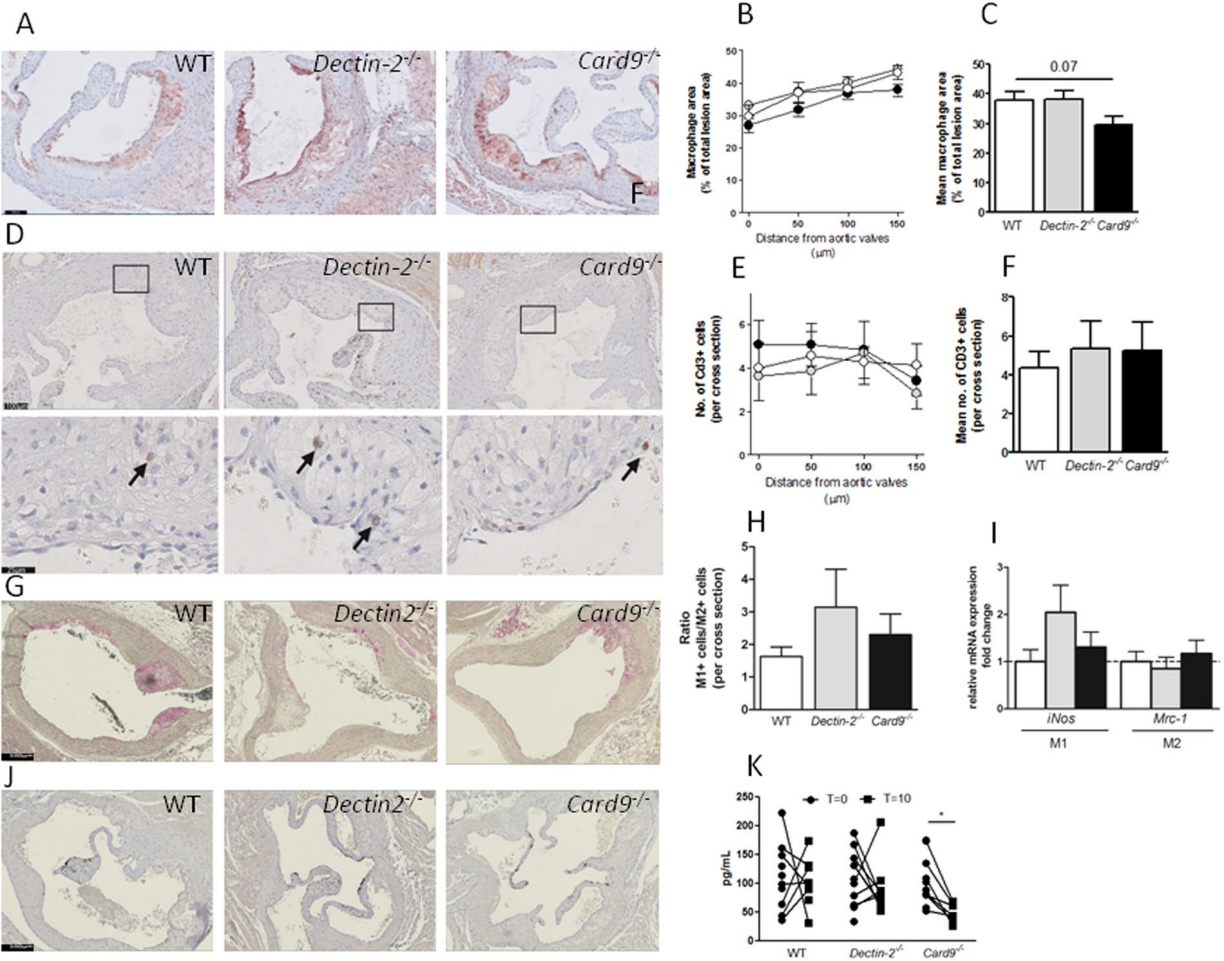
Hematopoietic deletion of CARD9, decreased plasma MCP-1 levels, tended to decrease macrophage content but does not altered the distribution of M1/M2 markers in the plaque. After 10 weeks of Western-type diet (WTD) feeding, hearts were collected from *Ldlr*^−/−^ mice transplanted with BM from control (WT), *Dectin-2*^−/−^ or *Card9*^−/−^ and aortic roots were analyzed by Immunohistochemistry. (**A**) Macrophage area was measured by MAC3 staining, (**B**) quantified in 4 sections and (**C**) average was calculated. T-cells were determined by CD3 staining, (**D**) counted in 4 sections and (**E**) average was calculated (**F**). M1 and M2 macrophages were determined by (**G**) iNos and (**J**) Arginase staining and (**H**) quantified in 12 sections and presented as ratio between M1/M2 marker. (**I**) RT-qPCR was used to quantify the expression of markers for M1 and M2 macrophages. (**K**) Plasma levels of monocyte attractant protein 1 (MCP-1) were measured at the beginning (T = 0) of WTD feeding and at the end of the study (T = 10). (**K**). Representative pictures for the staining are shown with  sclae bars representing 100 µm. Data for atherosclerotic plaque composition are presented as mean ± SEM. n = 13–14/group. Plasma MCP-1 levels. n = 7–11/group, *p < 0.05.

Deletion of hematopoietic Dectin-2 did not influence macrophage (Fig. 3A–C) or T-cell (Fig. 3D–F) content within the lesions. While deletion of hematopoietic CARD9 tended to reduce macrophage content within the lesion (Fig. 3A–C, *p* = 0.07), T-cell content within the lesions was not different (Figs 2H–J and 3D–F). Immunohistochemical staining for iNOS (M1 marker, Fig. 3G) and Arginase (M2 marker, Fig. 3J) revealed no differences in M1/M2 ratio among the three genotypes (Fig. 3H). Similar, gene expression in the remainder of the aorta was assessed to determine the distribution of M1 and M2 marker but *iNos* and *Mcr-1* (Fig. 3I) were not differentially expressed among the three genotypes. Interestingly, we found that deletion of hematopoietic CARD9 reduced plasma levels of MCP-1 upon WTD feeding (Fig. 3K). Collectively, our data do not support a role for Dectin-2 in atherosclerosis development, while deletion of hematopoietic CARD9 increased atherosclerosis development, but tended to reduce macrophage content in line with reduced monocyte recruitment signaling, without altering the phenotype of macrophages recruited to the aorta or plaque.

### Hematopoietic deletion of Dectin-2 or CARD9 did not influence immune cell composition in the circulation or in bone marrow

**Figure 4:**
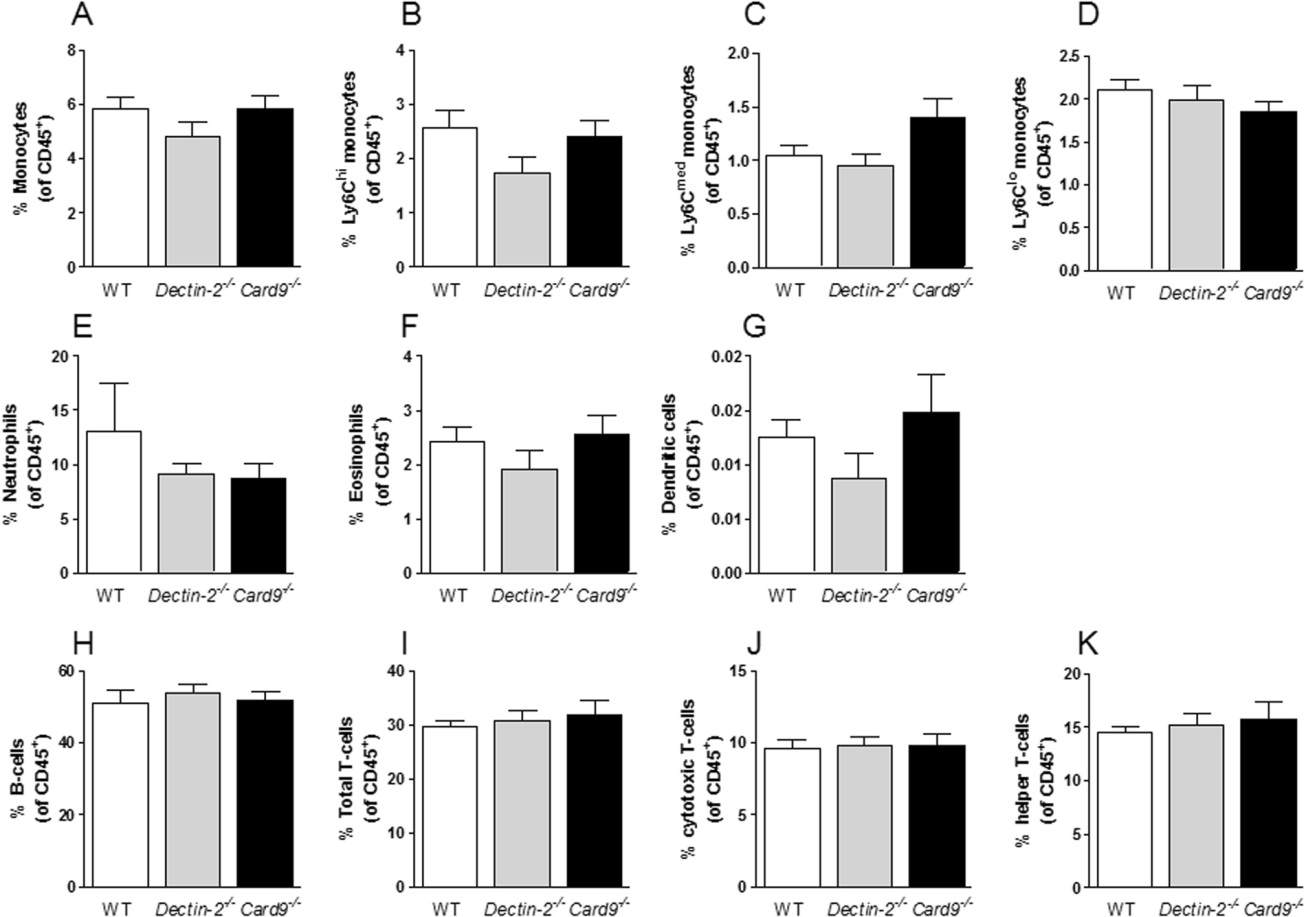
Hematopoietic deletion of Dectin-2 or CARD9 hardly affects immune cell composition in the circulation or in bone marrow. At the end of the study, after 10 weeks of Western-type diet feeding, immune cells subsets in the blood of *Ldlr*^−/−^ mice transplanted with BM from control (WT), *Dectin-2*^−/−^ or *Card9*^−/−^ were determined by flow cytometry. The amount of innate immune cell subsets are shown for (**A**) total Ly6C monocytes, which were subdivided into (**B**) Ly6C^hi^-, (**C**) Ly6C^med^-, (**D**) Ly6C^lo^-monocytes. Further, the percentage of (**E**) neutrophils, (**F**) eosinophils and (**G**) dendritic cells is shown. The percentage of adaptive immune cell subsets is determined for (**H**) B-cells, (**I**) total T-cells and T-cell subsets such as (**J**) cytotoxic T-cells and (**K**) T-helper cells. Data are presented as mean ± SEM. n = 7–8/group.

To determine whether the differential atherosclerotic plaque formation in mice with a deletion of hematopoietic Dectin-2 or CARD9 was founded by altered immune cells subsets in the circulation or in BM, FACS analysis was performed on leukocytes in whole blood (Fig. 4) and in BM (Supplementary Fig. 3). Innate immune cells such as monocytes (Fig. 4A), subsets of monocytes (*i.e*. Ly6C^hi^, Fig. 4B; Ly6C^med^, Fig. 4C; Ly6C^lo^, Fig. 4D), neutrophils (Fig. 4E), eosinophils (Fig. 4F) and dendritic cells (Fig. 4G) in blood were not altered by deletion of hematopoietic Dectin-2 or CARD9. Similarly, the number of adaptive immune cells such as B-cells (Fig. 4H) and total T-cells (Fig. 4I) and T-cell subsets (*i.e*.; cytotoxic T-cells, Fig. 4J; T-helper cells, Fig. 4K) in blood were comparable between all groups as shown by representative plots (Supplementary Fig. 3A,B). Also in the BM, the number of leukocyte subsets was not different between the groups (Supplement Fig. 3). We only observed an increased percentage of total T-cells in BM of mice with hematopoietic CARD9 deletion (Supplementary Fig. 3H).

### Deletion of hematopoietic Dectin-2 or CARD9 did not influence cytokine secretion

**Figure 5:**
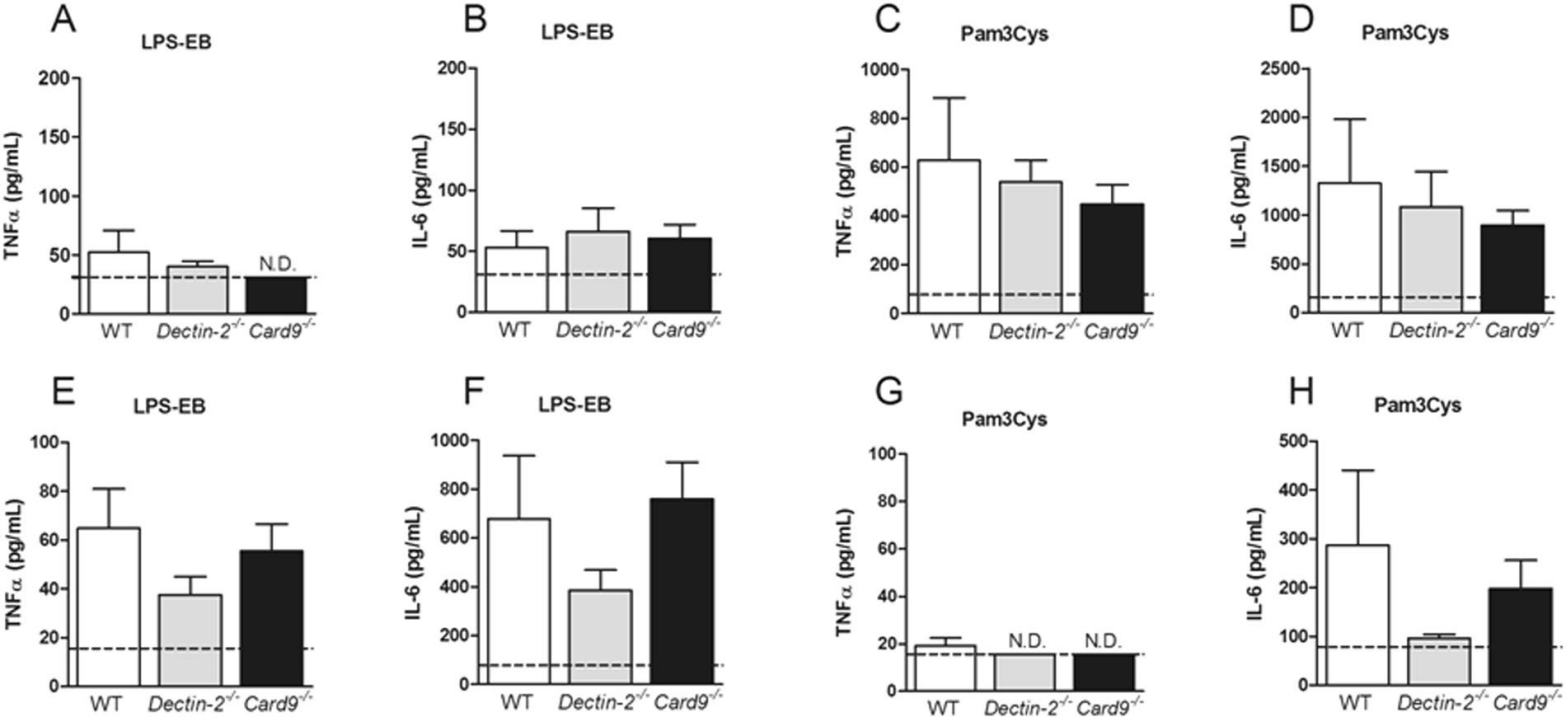
Deletion of hematopoietic Dectin-2 or CARD9 does not influence cytokine secretion. At the end of the study, after 10 weeks of Western-type diet feeding bone marrow-derived macrophages (BMDMs, **A**–**D**) and peritoneal cells (**E**–**H**) from *Ldlr*^−/−^ mice transplanted with BM from control (WT), *Dectin-2*^−/−^ or *Card9*^−/−^ were isolated and *ex vivo* stimulated. (**A**) Tumor necrosis factor (TNF)α and (**B**) Interleukin (IL-)6 cytokine secretion were measured after stimulation with (**A**,**B**) lipopolysaccharide (LPS-EB, 10 ng/mL) or (**C**,**D**) Pam3Cys (10 µg/mL) in BMDMs. Similarly, (**E**,**G**) TNFα and (**F**,**H**) IL-6 were measured after stimulation with (**E**,**F**) LPS-EB or (**G**,**H**) Pam3Cys. Data are presented as mean ± SEM. n = 8/group. N.D., values are below detection limit. Detection limit is indicated with the black dotted line.

As the composition of immune cells in the circulation or BM could not explain the differential atherosclerotic lesion formation in mice with a hematopoietic deletion of Dectin-2 or CARD9, the functionality of isolated peripheral immune cells, splenocytes, BMDMs and peritoneal cells were evaluated by performing *ex vivo* stimulations. As expected, deletion of hematopoietic CARD9 reduced the TNFα secretion from *Candida albicans*-stimulated splenocytes, confirming impaired cytokine response upon CLR specific stimulation (Supplementary Fig. 4). Deletion of hematopoietic Dectin-2 did not significantly reduce TNFα secretion by splenocytes in response to *Candida albicans* (Supplementary Fig. 4). BMDMs and peritoneal cells were stimulated with various stimuli, including the TLR4 ligand LPS-EB and the TLR2 ligand Pam3Cys, to assess the general (CLR independent and CLR-TLR codependent) capacity of these cells to secrete cytokines (Supplementary Fig. 5B–F). Deletion of hematopoietic Dectin-2 or CARD9 neither influenced secretion of IL-6 (Fig. 5B,D) nor TNFα (Fig. 5A, Supplementary Fig. 4B–F) from stimulated BMDMs nor secretion of IL-6 (Fig. 5F,H) or TNFα (Fig. 5E) from stimulated peritoneal cells (Fig. 5E,F,H). The IL-6 secretion by BMDMs upon LPS-EB stimulation as well as the TNFα secretion upon P3C stimulation by peritoneal cells was beyond detection limit of the assay (5A,G).

## Discussion

Innate immune cells are crucial for atherosclerotic plaque development and can be activated by PRRs. The impact of the CLR family of PRRs, however, remained largely unclear. The aim of this study was to evaluate the role of Dectin-2, as well as CLR signalling via CARD9, on inflammation and atherosclerosis development in *Ldlr*^−/−^ mice. Our data show that deletion of hematopoietic Dectin-2 neither affected plasma lipid levels nor the immune cell composition and *ex vivo* cytokine secretion. In addition, atherosclerotic lesion size and composition were not affected by deletion of hematopoietic Dectin-2. Deletion of hematopoietic CARD9 did not influence plasma lipids, circulating immune cell composition and peripheral cytokine secretion, but surprisingly promoted plaque formation.

Our results in the absence of hematopoietic Dectin-2 deficiency are in line with a mouse model of myocardial infarction, in which immune cell recruitment and macrophage polarization were not altered in Dectin-2-deficient mice after permanent coronary ligation^16^. Thus, although monocytes are among the first cells to arrive at the site of lipid accumulation during atherosclerotic lesion formation^12^ and macrophages express high levels of Dectin-2, our findings do not provide evidence for a role of hematopoietic Dectin-2 in atherosclerotic lesion development under hyperlipidemic conditions^12^. Even though hematopoietic deletions of either Dectin-1^10^ or Dectin-2 have not shown to impact on atherosclerotic plaque formation, given their redundancy in fungal recognition, it cannot be excluded that there are effects on the development of atherosclerosis in a model with a hematopoeitic deletion of both receptors.

Two reasons may account for the absence of any effects of deletion of hematopoietic Dectin-2 in our study. First, there could be a functional redundancy for individual CLRs, meaning that CLRs cooperate in a coordinated response and absence of a single receptor will be compensated for by others^17^, a phenomena that has been described in the response to pathogens, especially *in vivo*^8,17^. Second, the absence of any endogenous ligands for Dectin-2 in the circulation or atherosclerotic lesions of the *Ldlr*^−/−^ mice may explain our findings. In general, CLRs are characterized by a carbohydrate recognition domain which recognizes carbohydrate structures on pathogen-derived exogenous ligands, such as fungal wall components^18,19^. However, although studies reveal the involvement of this receptor in fungal recognition, no specific ligand could be identified up to now^20^. Accumulating evidence suggest that CLRs can also recognize a variety of endogenous ligands including proteins, lipids and cholesterol crystals derived from *e.g*. necrotic cells^11,19,21^. In addition, Dectin-2 has been proposed to recognize damage-associated molecular patterns (DAMPs) released by necrotic cells^16^. However, specific endogenous relevant during atherosclerotic development, have not yet been identified for Dectin-2.

The second objective of the current study was to examine the role of downstream CLR signalling via CARD9 in atherosclerosis. CARD9 is expressed in various innate immune cells and mediates cytokine secretion. Inhibition of CARD9 has therefore been hypothesized as an interesting therapeutic strategy to reduce inflammation related cardiovascular pathologies such as atherosclerosis^13^. However, our data showed that deletion of hematopoietic CARD9 even increased the atherosclerotic lesion size and lesion severity in *Ldlr*^−/−^ mice. This is surprising because hematopoietic deletion of several CLRs that signal via CARD9 so far yielded either no effect on atherosclerosis (*i.e*. Dectin-1^10^ and Dectin-2 in the current study) or a protection from atherosclerotic lesion development (*i.e*. Mincle^11^) in *Ldlr*^−/−^ mice. The reduction in atherosclerotic lesion development upon hematopoietic deletion of Mincle was observed after prolonged WTD feeding (*i.e*. 20 weeks), which coincides with extremely severe atherosclerotic lesions that contain a very high amount of necrotic cells^11^. Since necrotic cells are believed to provide endogenous ligands for Mincle^11,22^ and have been shown to activate CARD9 signaling^15^, one might speculate that deletion of CARD9 reduces lesion development in a model with advanced lesions as apparent in our study. Instead, hematopoietic deletion of CARD9 led to potentially less stable and lipid-rich plaques. In fact, upon WTD-feeding plasma MCP-1 levels dropped in mice with a deletion of hematopoietic CARD9 leading to a lower number of macrophages in the plaque. It can be speculated that this explains the more severe plaque phenotype and the increased plaque area in mice with a deletion of hematopoietic CARD9.

Our results show that hematopoietic CARD9 deletion did not influence the number of circulating immune cell types, or the inflammatory phenotype of peripheral immune cells. However, we found that deletion of hematopoietic CARD9 tended to reduce lesion macrophage area which was accompanied by the reduced levels of plasma MCP-1. Our data show less profound effects on the inflammatory state compared to a previous study that found protective effects of whole body deletion of CARD9 on inflammation-driven neointima formation of grafted veins^15^. In this inflammatory model, CARD9 deletion reduced the recruitment of monocytes into grafted veins and decreased mRNA expression of the pro-inflammatory cytokines *Il-1β*, *Il-6* and *Mcp-1* on infiltrating macrophages^15^. Importantly, CARD9 deletion not only reduces macrophage infiltration and inflammatory cytokine secretion in this high-inflammatory vein graft model, but also in another disease model driven by low-grade inflammation. Specifically, whole-body *Card9*^−/−^ mice were protected from high-fat diet-induced macrophage infiltration in the heart and myocardial dysfunction^23^. Moreover, in this obesity model, CARD9 deletion reduced IL-6 and IL-1β levels in plasma as well as pro-inflammatory cytokine secretion from isolated peritoneal cells^23^. These findings were established in whole-body *Card9*-knockout mice and oppose our findings in mice with CARD9 deletion only in the hematopoietic compartment. Therefore, it is possible CARD9 expression in other cell types than immune cells induces macrophage infiltration in low- and high grade inflammatory conditions. We anticipate that in our study, CARD9 expression in other cell types counteracted the otherwise potentially atheroprotective effects of reduced levels of MCP-1^24^ found in mice with a deletion of hematopoietic CARD9. For example, CARD9 expression has been observed in endothelial cells *in vitro* under conditions of shear stress^25^ and shear stress affects macrophage infiltration and polarization *in vivo* in atherosclerotic plaques^26^. It is therefore possible that activation of CARD9 in non-immune cells such as endothelial cells contributes to inflammatory processes under metabolically disturbed conditions. Another difference with the obesity model is the presence of high plasma glucose levels in obesity due to high-fat diet-induced glucose intolerance. As mentioned above, CLRs are classically involved in the recognition of carbohydrate structures on pathogen-derived exogenous ligands^19^. It may be feasible that, under hyperglycemic conditions, glycosylated protein (sugar-like) structures are formed that function as ligands for CLRs, leading to immune cell activation and macrophage infiltration. In this scope, future studies should determine whether CARD9 deletion reduces macrophage infiltration and atherosclerosis development under hyperglycaemic conditions.

Of note, macrophage area tended to be reduced in atherosclerotic lesions upon hematopoietic deletion of CARD9 in our study, but the phenotype of the macrophages did not differ. Upon infiltration into the lesion, monocytes differentiate into macrophages thereby phenotypically adapting to the encountered environment^1^. However, in our study we showed that the ratio of classical markers for M1 and M2 is not different in the plaque among the three genotypes. More indirect measures of lesion macrophage phenotype, such as cytokine secretion of peripheral macrophages and expression of inflammatory markers in the aorta and the liver confirmed these findings. Similar, although the process of macrophage infiltration in livers of WTD-fed *Ldlr*^−/−^ mice shows similarities to atherosclerotic lesions^27^, specific liver mRNA expression may only to some extent represent processes that might have taken place in the atherosclerotic lesion^27^. Moreover, it needs to be noticed that our study was performed in female mice. According to a study that evaluated the impact on gender on plaque formation in LDLr^−/−^ mice using similar amounts of cholesterol in their WTD^28^, female mice develop significantly bigger atherosclerotic lesions than male mice. Therefore, if anything, we would expect an even more pronounced effect of hematopoietic CARD9 deficiency compared to WT using male mice.

Moreover, different genetic approaches of inhibiting hematopoietic transcription factor NF-κB, a key regulator of inflammation via TLRs and CLRs recognition, did not only attenuate^29^ but also promoted^30^ atherosclerotic plaque formation indicating its crucial role in both pro- and anti-inflammatory processes in atherosclerosis. Similar, deletion of CARD9 in our model may have disrupted a status of balanced pro- and anti-inflammatory signaling ultimately leading to increased plaque formation. It can be speculated that CARD9 coordinates responses to lipid triggers eventually leading to differential recruitment of monocytes and to morphological and dimensional unfavorable plaques.

In summary, our results do not support a fundamental role for Dectin-2 in inflammation or atherosclerotic lesion development, while deletion of hematopoietic CARD9 promotes atherosclerotic plaque development in hyperlipidemic *Ldlr*^−/−^ mice.

## Methods

### Animals

Female homozygous *Ldlr*^−/−^ mice (background C57Bl/6J) were obtained from Jackson Laboratory (Bar Harbor, ME, USA). Mice were housed under standard conditions in conventional cages. To induce BM aplasia, *Ldlr*^−/−^ recipient mice were randomized based on age, body weight and plasma lipid levels and exposed to a single dose of 8 Gy using an X-RAD (RPS Services Limited, Surrey, UK). The day thereafter irradiated recipient *Ldlr*^−/−^ mice received an intravenous injection via the tail vein with 1.2 × 10^6^ BM cells isolated from donor control C57Bl/6J WT, *Dectin2*^−/−^ or *Card9*^−/−^ female mice (all C57Bl/6J background), mixed with 0.3 × 10^6^ freshly isolated splenic cells from *Rag1*^−/−^ female mice (C57Bl/6J background). From one day before until 4 weeks after BM transplantation, all mice received water, containing antibiotics (0.13 mg/kg/day Ciprofloxacin, 0.105 mg/kg/day Polymyxin B, 0.15 mg/kg/day Amfotericine B). After 10 weeks of recovery on chow diet, mice received WTD containing 15% (w/w) cocoa butter, 1% (w/w) corn oil and 0.1% (w/w) cholesterol (AB diets, Woerden, The Netherlands) for 10 weeks (Fig. 1A). At the end of the study mice were killed and various organs and tissues were collected. Successful deletion of hematopoietic Dectin-2 and CARD9 was confirmed in the bone marrow (data not shown). All experiments in this study were approved by the Ethics Committee on Animal Experiments of the Leiden University Medical Center and performed in accordance with relevant guidelines and regulations.

### Plasma lipid and systemic inflammation analysis

At the indicated time points, mice were fasted for 4 h and blood was collected via the tail vein. After 10 weeks of WTD, unfasted blood samples were collected via orbital exsanguination in EDTA-coated tubes. Plasma from all samples was isolated by centrifugation and assayed for total cholesterol and triglycerides using commercially available enzymatic colorimetric kits (Liquicolor, Human GmbH, Wiesbaden, Germany). Assays were performed according to the manufacturer’s protocols.

### Atherosclerosis quantification

Hearts were collected and fixed in phosphate-buffered 4% formaldehyde, embedded in paraffin and cross-sectioned (5 µm) throughout the aortic root area, starting from the appearance of open aortic valve leaflets. Per mouse, four sections with 50-μm intervals were used for atherosclerosis quantification. Sections were stained with hematoxylin-phloxine-saffron for histological analysis. Macrophage area was determined using Rat anti-mouse antibody MAC3 (1:1000; BD). Sections were incubated with a rabbit polyclonal antibody directed against CD3 (1:50; DakoCytomation, Glostrup, Denmark) to identify CD3 T lymphocytes. The lesion area and composition were quantified using ImageJ Software.

Macrophage markers for inducible nitric oxid syntase (iNOS, 1:200, Abcam, UK) and Arginase (1:500, Thermo Fisher Scientific) were used to determine the amount of M1 and M2 macrophages, respectively. The abundance of M1/M2 markers were counted per section by one blinded researcher and is presented as ratio of the mean.

Atherosclerotic lesions were categorized for severity by one blinded observer, according to the guidelines of the American Heart Association, adapted for mice. Lesions were classified into various types: type 0 (no lesions), type I to II (early fatty streak-like lesions containing foam cells in the media, and type IV-V (advanced lesions containing foam cell in the media, presence of fibrosis, and cholesterol clefts, mineralization, and/or necrosis).

### Cytokine- and Quantikine- assay

The concentrations of mouse TNFα (R&D Systems, Minneapolis, MN) and mouse IL-6 (Sanquin, Amsterdam, Netherlands) were measured in the cell culture supernatants using enzyme-linked immunosorbent assays (ELISAs). The concentration of mouse monocyte chemoattractant protein (MCP)-1 (R&D Systems) was measured in plasma samples at the start (T = 0) and after 10 weeks of WTD feeding at the end of the study (T = 10) using ELISA. All assays were preformed according to the manufacturer’s instructions.

### RNA Isolation and qPCRs

Trizol reagent (Invitrogen, Carlsbad, CA, USA) was used according to manufacturer’s protocol to extract aortic and liver mRNA (Supplemental Fig. 5) which was then transcribed into complementary DNA (cDNA) by reverse-transcription using iScript cDNA synthesis kit (Bio-Rad Laboraties BV, Veenendaal, The Netherlands). Relative expression was determined using SYBR Green method (Applied Biosystem, Thermo Fisher Scientific) on an Applied Bioscience Step-one PLUS qPCR machine (Applied Biosystems, Life technologies, Thermo Fisher Scientific) and the values were expressed as fold increases in mRNA levels relative to those of mice receiving WT bone marrow. *36b4* was used as a housekeeping gene. Primers used for the experiments (final concentration 10 µM) are listed in Table S1.

### FACS analysis

Fifty µL fresh blood was stained with antibodies for CD45, SiglecF, Ly6C, CD4 (BD Bioscience, Breda, The Netherlands); CD11b, Ly6G, MHCII, CD3, CD8a, NK (Biolegend, San Diego, CA, USA); and CD19 (eBioscience, Thermo Fisher Scientific, Breda, The Netherlands). Staining was analyzed by FACS (FACS Verse; BD Bioscience) and CXP software (Beckman Coulter, Woerden, The Netherlands). Whole blood was first gated on total CD45^+^ leukocyte population. Neutrophils were select as Ly6G^+^, and within the Ly6G^−^ population eosinophils were defined as CD11b^+^-SiglecF^+^. Within the Ly6G^−^SiglecF^−^ population, monocytes were selected by excluding CD11b^+^MHCII^+^ dendritic cells. Monocytes were then defined as Ly6C^high(hi)^ reflecting pro-inflammatory monocytes, Ly6C^low(lo)^ reflecting anti-inflammatory monocytes, or Ly6C^medium(med)^ reflecting monocytes with an intermediate inflammatory phenotype. Lymphoid cells were selected on CD19^+^ for B-cells and CD3^+^ for T-cells. Within CD3^+^ population cytotoxic T-cells were gated on CD8^+^CD4^−^ and T-helper cells on CD8^−^CD4^+^.

BM was harvested in cold PBS and BM suspension was passed through 70-µm nylon mesh (BD Biosciences). Lineage depletion was performed for the hematopoietic stem and progenitor cell (HSPC) analysis by magnetic bead isolation according to the manufacturer’s instructions (Lineage Cell Depletion Kit; Miltenyi Biotec, Teterow, Germany). BM cell suspensions were incubated in hypotonic lysis buffer (8.4 g NH_4_Cl and 0.84 g NaHCO_3_ per liter distilled water) to remove erythrocytes. Mature BM cells but not HSPCs were incubated with an Fc-receptor blocking antibody (Fc block, eBioscience, Thermo Fisher Scientific) to prevent non-specific binding. Mature cell suspension was extracellularly labeled with antibodies for CD45 (Biolegend); Ly6C (AbD serotec, Oxford, UK); CD3, CD19 (eBioscience); Ly6G, and CD11b (BD Pharmigen, San Diego, CA, USA). Staining was analyzed by FACS (FACSCanto II, BD Bioscience) and FlowJo software version 7.6.5 (Ashland OR, USA).

### *Ex vivo* cytokine stimulations

For *ex vivo* cell stimulation experiments, cells were extracted from the spleen, the BM (*i.e*. tibia and femur of hind limb bones) or the peritoneum. Splenocytes were obtained by crushing whole spleen through 70 µm nylon mesh (Corning, Amsterdam, The Netherlands) with a plunger of a 1 mL syringe (BD Plastipak) and fat particles were filtered out. Cell suspension was spun down and taken up in RPMI 1640 supplemented with 50 µg/mL Gentamicin, 1 mM Pyruvate and 2 mM glutamax and 10% heat inactivated foetal bovine serum (Life Technologies, Thermo Fisher). Four hundred µL cell suspension of 5 × 10^6^ splenocytes were added to 24 well round-bottom (Corning) plates and stimulated with *Candida albicans* (1 × 10^7^/mL), For extraction of BM cells, bones were cleaned with 70% ethanol, cut and flushed with sterile phosphate buffered saline (PBS). Obtained cells were then differentiated in Dulbecco’s Modified Eagle’s medium (DMEM, Thermo Fisher Scientific) containing 1% Penicillin/Streptomycin (Sigma-Aldrich, St. Luis, MO, USA) and 30% (vol/vol) L929 medium for 7 days. Then cells were counted with particle counter (Beckmann Coulter). For stimulation experiments, 100 µL cell suspension of 1 × 10^5^ bone marrow-derived macrophages (BMDM) were added to 96-well flat bottom plates (Corning) and stimulated with *Escherichia coli* (*E. coli*) lipopolysaccharide (LPS-EB, 10 ng/mL) (serotype O55:B5; Sigma-Aldrich, St. Louis, MO, USA), Pam3Cys (10 µg/mL, EMC Microcollections, Tübingen, Germany), *Mycobacterium tuberculosis* (H37Rv, 5 µg/mL), poly I:C (50 µg/mL, Invivogen, San Diego, CA, USA), Candida *albicans (*1 × 10^7^/mL)*, Staphylococcus aureus (*HK, 1 × 10^7^/mL), and *Salmonella typhimurium (*HK, 1 × 10^7^/mL). Peritoneal cells were obtained by injecting 10 mL ice-cold PBS into the peritoneal cavity. Total cavity fluid was collected, spun down and obtained cells were counted and resuspended in RPMI culture medium (MP Biomedicals, Santa Ana, CA, USA) supplemented with 1 mM pyruvate, 50 µg/mL gentamycin (Life TechnologiesThermo Fisher Scientific), 1 mM HEPES and 5.5 mM D-glucose (Sigma-Aldrich). Hundred µL cell suspension of 1 × 10^5^ peritoneal cells was added to 96 well round-bottom plates (Greiner, Monroe, North Carolina, USA) and stimulated with LPS-EB (10 ng/mL) or Pam3Cys (10 µg/mL). Supernatants of stimulated splenocytes were collected after 48 hours and supernatants of BM cells and peritoneal cells were collected after 24 hour and stored at −80 °C until analyses.

### Statistical analysis

Data are shown as means ± standard error of the mean (SEM). Differences in cytokine secretion were tested using the paired Wilcoxon ranked test. Differences in atherosclerotic lesion size, blood and BM cell populations were determined using one-way ANOVA followed by Dunnetts post-hoc test. Differences at probability values less than 0.05 were considered statistically significant. All statistical analyses were performed in Graphpad Prism 5.

## Supplementary information

Supplementary info

## Data Availability

All data generated or analysed during this study are included in this published article (and its Supplementary Information files).
