# Supplementary material for: Deletion of hematopoietic Dectin-2 or CARD9 does not protect against atherosclerotic plaque formation in hyperlipidemic mice
Source: Sci Rep. 2019 Mar 13;9:4337. doi: 10.1038/s41598-019-40663-x (PMC6416398; doi:10.1038/s41598-019-40663-x)
Supplement: Supplementary file 1 — Supplementary info [file 41598_2019_40663_MOESM1_ESM.pdf]

Deletion of hematopoietic Dectin-2 or CARD9 does not protect against atherosclerotic plaque formation in hyperlipidemic mice

Kathrin Thiem[1,#,*], Geerte Hoeke[2,3,#], Susan van den Berg[7], Anneke Hijmans[1], Cor W.M. Jacobs[1], Enchen Zhou[2,3], Isabel M. Mol[2,3], Maria Mouktaroudi[4], Johan Bussink[5], Thirumala D. Kanneganti[6], Esther Lutgens[7,8], Rinke Stienstra[1,9], Cees J. Tack[1], Mihai G. Netea[1,10], Patrick C.N. Rensen[2,3], Jimmy F. P. Berbée[2,3,$], Janna A. van Diepen[1,$]

[1]Dept. of Internal Medicine and Radboud Institute for Molecular Life Sciences, Radboud university medical center, Nijmegen, The Netherlands; [2]Dept. of Medicine, Div. of Endocrinology, [3]Einthoven Laboratory for Experimental Vascular Medicine, Leiden University Medical Center, Leiden, The Netherlands; [4]Dept. of Internal Medicine, National and Kapodistrian University of Athens, Medical School, Athens, Greece; [5]Dept. of Radiation Oncology, Radboud University Medical Center, Nijmegen, The Netherlands; [6]Department of Immunology, St. Jude Children's Research Hospital, Memphis, TN, USA; [7]Dept of Medical Biochemistry, Div. of Experimental Vascular Biology, Academic Medical Center, University of Amsterdam, Amsterdam, The Netherlands; [8]Institute for Cardiovascular Prevention, Ludwig Maximilians University of Munich, Munich, Germany; [9]Div. of Human Nutrition, Wageningen University, Wageningen, The Netherlands; [10]Dept. for Genomics & Immunoregulation, Life and Medical Sciences Institute (LIMES), University of Bonn, Bonn, Germany.

[#,$] Authors contributed equally.

*** Corresponding author:**
Kathrin Thiem, Department of Internal Medicine (463), Radboud university medical center, Geert Grooteplein zuid 8, 6525 GA, Nijmegen, The Netherlands
Phone: +31-243610244; E-mail: kathrin.thiem@radboudumc.nl

Supplementary Table 1: Primer pairs used for amplification of aortic and liver genes

		**Primer sequence 5'->3'**	
**Primer name**	**Gene accession numbers**	forward	reverse
Cd68	NM_001291058.1	CCAATTCAGGGTGGAAGAAA	CTCGGGCTCTGATGTAGGTC
iNos	1.NM_001313921.1	CGTTTCGGGATCTGAATGTGA	GGGCAGCCTGTGAGACCTT
Mcr-1	NM_008625.2	CTCTGTTCAGCTATTGGACGC	CGGAATTTCTGGGATTCAGCTTC
Abca1	NM_013454.3	GCTTGTTGGCCTCAGTTAAGG	GTAGCTCAGGCGTACAGAGAT
Abcg1	NM_009593.2	GTGGATGAGGTTGAGACAGACC	CCTCGGGTACAGAGTAGGAAAG
Cd68	NM_001291058.1	CCAATTCAGGGTGGAAGAAA	CTCGGGCTCTGATGTAGGTC
Cd11c	NM_001363985.1<br>NM_021334.3<br>NM_001363984.1	CTGGATAGCCTTTCTTCTGCTG	GCACACTGTGTCCGAACTCA
Clec5a	NM_021364.2<br>NM_001038604.1	TGTGTTCAATGGCAATGTTACCA	GCAGATCCAGCGATAGCTGAC
Clec4f	NM_016751.3	ACTGAAGTACCAAATGGACAATGTTAGT	GTCAGCATTCACATCCTCCAGA
Dectin-1	NM_020008.3,<br>NM_001309637.1	AGGTTTTTCTCAGCCTTGCCTTC	GGGAGCAGTGTCTCTTACTTCC
F4/80	NM_001355722.1<br>NM_001355723.1	CTTTGGCTATGGGCTTCCAGTC	GCAAGGAGGACAGAGTTTATCGTG
Gata3	NM_001355111.1<br>NM_001355112.1<br>NM_001355110.1<br>NM_008091.3	AAGCTCAGTATCCGCTGACG	GTTTCCGTAGTAGGACGGGAC
IL-6	NM_001314054.1<br>NM_031168.2	CAAGTCGGAGGCTTAATTACACATG	ATTGCCATTGCACAACTCTTTTCT
IL-1β	NM_008361.4	GCAACTGTTCCTGAACTCAACT	ATCTTTTGGGGTCCGTCAACT
Mcp-1	NM_011333.3	CCCAATGAGTAGGCTGGAGA	TCTGGACCCATTCCTTCTTG
MhcII	NM_207105.3<br>NM_001033978.3	AGCCCCATCACTGTGGAGT	GATGCCGCTCAACATCTTGC
Rorc	NM_001293734.1<br>NM_011281.3	TCCACTACGGGGTTATCACCT	AGTAGGCCACATTACACTGCT
Tgfb1	NM_011577.2	CCACCTGCAAGACCATCGAC	CTGGCGAGCCTTAGTTTGGAC
Tnf-α	NM_013693.3	CAGACCCTCACACTCAGATCATCT	CCTCCACTTGGTGGTTTGCTA
36b4	NM_001357772.1<br>NM_001277982.2<br>NM_001357768.1<br>NM_001357770.1<br>NM_001277986.1<br>NM_001277983.1<br>NM_013669.2	AGCGCGTCCTGGCATTGTGTGG	GGGCAGCAGTGGTGGCAGCAGC

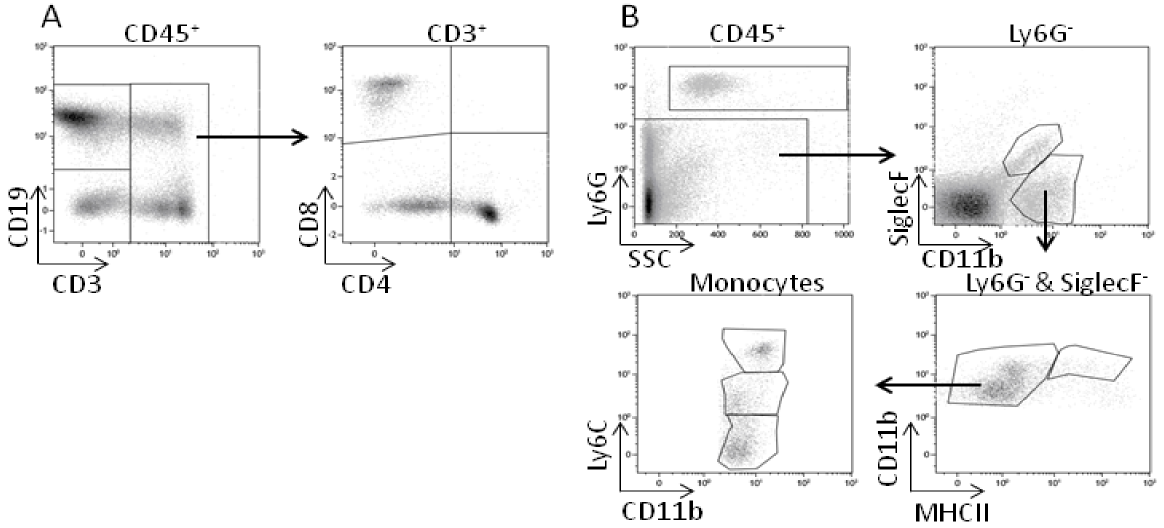

Supplementary Figure 2. Representative FACS plot for the identification of lymphoid and myeloid subsets in *Ldlr*$^{-/-}$ mice. (A) To identify lymphoid cells, B-cells (CD19^{+}) were excluded from innate immune cells (CD45^{+}), T-cells (CD19^{+} and CD3^{+}) were further subdivided into cytotoxic T-cells (CD8^{+}) and T-helper cells (CD4^{+}). To identify myeloid cells, (B) neutrophils (Ly6G^{+}) were excluded from innate immune cells (CD45^{+}) and non-neutrophils (Ly6G^{-} and SiglecF^{-}) cells were further subdivided into monocytes (CD11b^{+}). Monocytes were then classified into pro-inflammatory (Ly6C^{high}), intermediate (Ly6C^{medium}) and anti-inflammatory (Ly6C^{low}) monocytes.

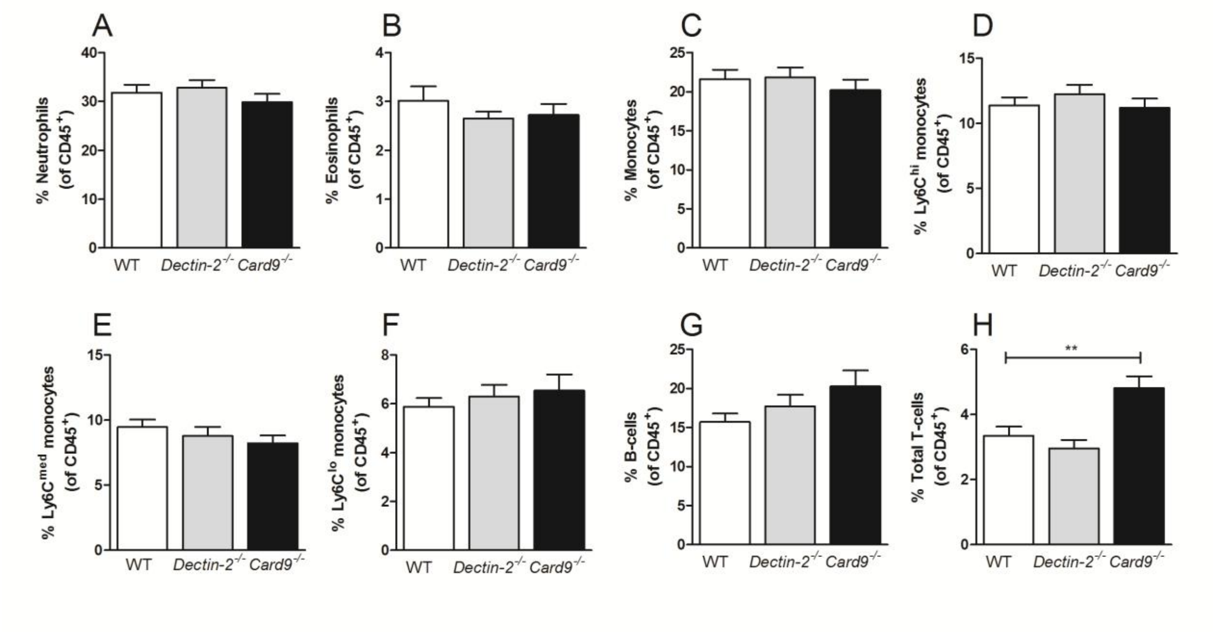

Supplementary Figure 3. Depletion of CARD9 increases T-cell percentage in the bone marrow. At the end of the study, after 10 weeks of Western-type diet feeding immune cell subset in *Ldlr*$^{-/-}$ mice transplanted with bone marrow from control (WT), *Dectin-2*$^{-/-}$ and *Card9*$^{-/-}$ mice were determined by flow cytometry. The percentage (of CD45^{+}) of innate immune cell subsets are shown for (A) neutrophils, (B) eosinophils, (C) total Ly6C monocytes and subsets such as (D) L6C^{hi}-, (E) Ly6C^{med}-, (F) Ly6C^{lo}-monocytes. The amount of adaptive immune cell subsets is shown for (G) B-cells and (H) T-cells. Data are presented as mean ± SEM. n=7-8/group. ***p*<0.01.

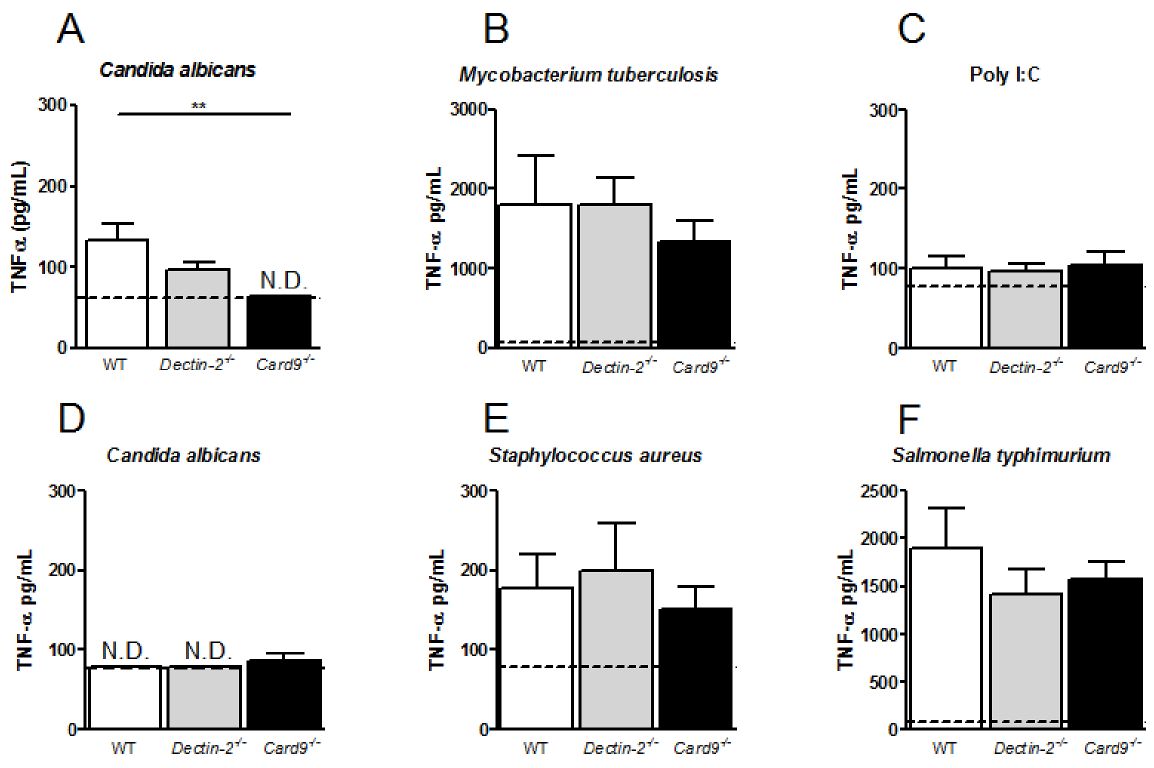

Supplementary Figure 4: Deletion of hematopoietic CARD9 reduces TNFα secretion from splenocytes but not from BMDMs upon stimulation with CLR or TLR ligands. At the end of the study, after 10 weeks of WTD feeding, mice were killed and splenocytes were isolated and *ex vivo* stimulated with (A) *Candida albicans.* Bone marrow-derived macrophages (BMDMs) were stimulated *ex vivo* with (B) *Mycobacterium tuberculosis* (H37Rv), (C) poly I:C, (D) *Candida albicans,* (E) *Staphylococcus aureus*, and (F) *Salmonella typhimurium.* The secretion of tumor necrosis factor (TNF)α by both cell types was determined. Data represent mean ± SEM. n=8/group. ***p*<0.01. Black dotted line indicates detection limit of the assay.

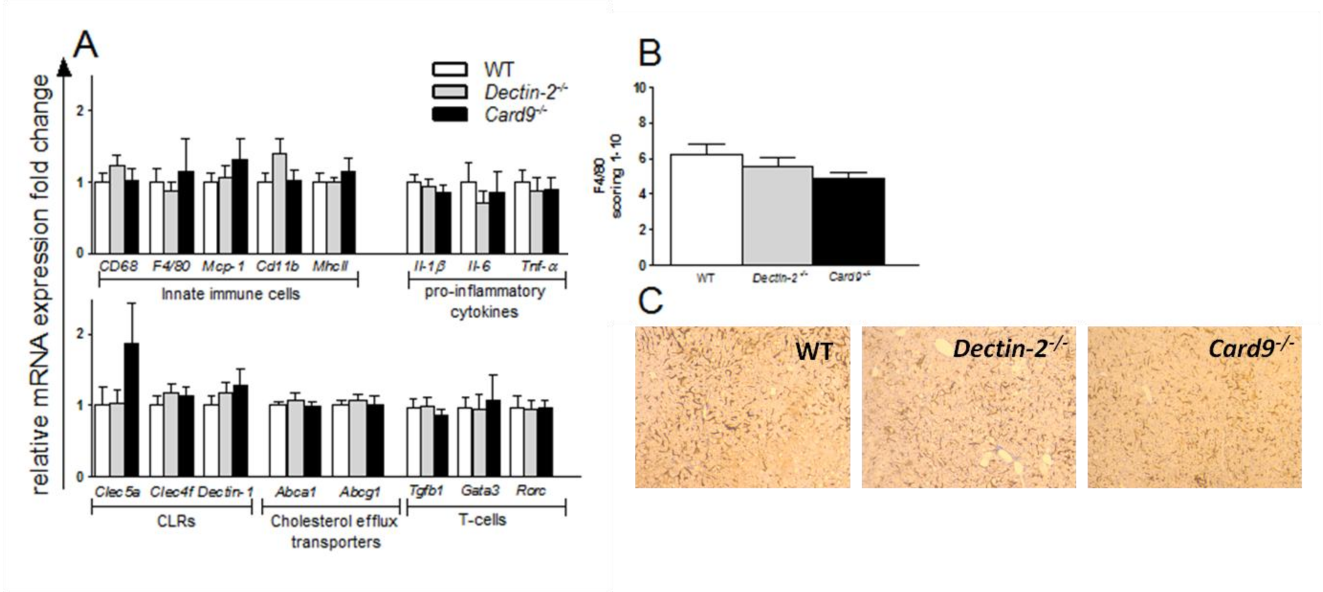

Supplementary Figure 5: Deletion of hematopoietic Dectin-2 or CARD9 did not influence hepatic expression of inflammatory markers or immunohistochemical macrophage marker. After 10 weeks of Western-type diet feeding, liver mRNA was isolated from *Ldlr*$^{-/-}$ mice transplanted with BM from control (WT), *Dectin-2*$^{-/-}$ or *Card9*$^{-/-}$ and RT-qPCR was used to quantify the (A) expression of markers for monocyte/macrophage, inflammation, C-type lectin receptors (CLRs), cholesterol metabolism and T-cells. Liver sections were prepared for histopathological analysis of macrophage marker F4/80 under a light microscope. (B) Macrophage content was ranked by two blinded researchers with a value between 1 and 10 whereby 10 is indicating greatest abundance. (C) Representative pictures of the liver are shown, x10. Data are presented as mean ± SEM. Gene expression: n=12/group, Immunohistochemical F4/80 staining: n=8/group. *Cd68*, Cd68 antigen; *F4/80*, Adhesion G protein-coupled receptor E1; *Mcp1*, Chemokine (C-C motif) ligand 2; *Cd11b*, Integrin alpha M; *MhcII*, Histocompatibility-2; *IL-1*β, Interleukin 1β; *IL-6,* Interleukin 6; *TNF*α, tumor necrosis factor α; *Clec5a*; CLR domain family 5 member A; *Clec4f,* CLR domain family 4 member F; *Dectin1/Clec7a*, CLR domain family 7 member A; *Abca1;* ATP binding cassette sub family A, member 1; *Abcg1*, ATP binding cassette sub family G, member 1; *Tgfb1*, Transforming growth factor, beta 1; *Gata3*, GATA binding protein 3; *Rorc*, RAR-related orphan receptor gamma.
